# Dr. Charles H. Dubs' Patent Compound Union Screw Forceps. Dr. S. P. Hullihen's Root Forceps

**Published:** 1849-07

**Authors:** 


### ARTICLE II.

### *Dr. Charles H. Dubs' Patent Compound Union Screw Forceps. Dr. S. P. Hullihen's Root Forceps.*

Messrs. Editors:—

In your valuable Journal, the star miscellany of Dental Science the world over, April number of the present year, I have noticed a miscellaneous remark respecting Dr. S. P. Hullihen's "Compound Screw Forceps," which I think somewhat calculated to injure the reputation of Dr. Charles H. Dubs, of Natchez, the patented inventor of an improvement to said compound screw forceps, if not the original inventor of the compound use of the forceps and screw. The fact is, that neither Dr. Dubs nor Dr. Hullihen invented either the forceps or the screw; any claim as inventors which either of these gentlemen have, must be confined to the compound use of these two discovered instruments in tooth-drawing.

The language of the miscellaneous notice in the quarterly journal is, "In the Eureka, a record of mechanism, inventions, patents, science and news, for Wednesday, April 18th, we find an engraving and description of Dr. S. P. Hullihen's compound screw forceps, which, as would seem from the above named publication, was patented by C. H. Dubs of Natchez, Miss., Oct. 17th, 1848. Now this instrument was invented several years ago, by Dr. Hullihen, of Wheeling, Va." &c.

The drawing and specification of Dr. Dubs' instrument may be seen, as above stated, in the Eureka; and the drawing and specification of Dr. Hullihen's in Professor Harris' "Principles and Practice of Dental Surgery," p. 292.

Dr. Dubs expressly claims as follows, in his specification in his patent, number 5865: "*A combination of springs and notches on the shaft of the screw with the catch of the click, by means of which the screw affords additional power in extracting roots of teeth.*" A moment's examination of both instruments, either by the drawings or by the instruments, as I have them both before me at this present writing, will convince any one that the *spring*, *notches* and *click*, which Dr. Dubs has attached to the shaft of the screw as used by Dr. Hullihen, gives the only real power the screw has, other than boring into and filling the cavity of a decayed tooth. The spring, notches and click constitute the only contrivance by which the screw can be made to pull at the same moment with the beaks of the forceps.

In this matter there can be no earthly dispute, as the instruments show for themselves. Dr. Hullihen's forceps seize the shaft of the screw in their beaks, and bore the head of the screw into the carious tooth; the beaks then open and clasp the sides of the tooth, every bit of the power of extraction being in the beaks of the forceps, the screw being as passive as a wooden peg driven into the centre of the tooth, and utterly incapable of drawing at the same time with the forceps; but being drawn out to the full length of the shaft, may be used simply as a screw, the beaks of the forceps constituting its handle.

But in Dr. Dubs' invention the shaft of the screw is seized by the beaks, and the boring is made as in the other; but now comes the real use of the screw. A delicate double spring is attached to the shaft of the screw, with a click adapted to a range of notches, say eight in number, cut into the shaft of the screw, which holds the shaft stiff and fast at any requisite distance, so as to make it draw at the same instant, the beaks of the forceps close upon the tooth, and the draw is made with them. The screw does its duty simultaneously with the beaks of the forceps, or if the sides, both of them, or only one, of the teeth may be carious, so that one or both beaks of the forceps cannot be pressed home hard upon the sides of the tooth, the pressure may be made just enough to hold the tooth together, while the whole power of extraction is entrusted to the screw. Any philosophical and practical dentist will in a moment see the superiority of the instrument invented by Dr. Dubs.

The writer of this communication, Messrs. Editors, has no interest or connection in this matter, other than a wish that the case should be understood, and that no injustice should be done Dr. Dubs. He has had, and will have, no part in any quarrel about the matter. Dr. Dubs, although qualified by his early education and business for a splendid instrument-maker, does not make any of his "Compound Union Screw Forceps" for sale. His mode of disposing of his improvement is to sell the right of making and individually using it in practice, to any dental surgeon for three dollars, as a life interest. F.

NATCHEZ, 26*th June*, 1849.

